# Peptide hydrogel *in vitro* non‐inflammatory potential

**DOI:** 10.1002/psc.2940

**Published:** 2016-12-19

**Authors:** A. Markey, V. L. Workman, I. A. Bruce, T. J. Woolford, B. Derby, A. F. Miller, S. H. Cartmell, A. Saiani

**Affiliations:** ^1^ School of MaterialsUniversity of ManchesterOxford Road M13 9PLManchesterUK; ^2^ Manchester Institute of BiotechnologyUniversity of ManchesterOxford Road M13 9PLManchesterUK; ^3^ Paediatric ENT Department, Royal Manchester Children's HospitalCentral Manchester University Hospitals NHS Foundation TrustManchester Academic Health Science CentreManchesterUK; ^4^ Division of Infection, Immunity and Respiratory Medicine, School of Biological Sciences, Faculty of Biology, Medicine and HealthUniversity of ManchesterOxford RoadM13 9PLManchesterUK; ^5^ Department of Otolaryngology–Head & Neck Surgery, Manchester Royal InfirmaryUniversity of ManchesterOxford RoadManchesterM13 9WL; ^6^ School of Chemical Engineering and Analytical ScienceUniversity of ManchesterOxford RoadM13 9PLManchesterUK

**Keywords:** peptide, hydrogel, monocytes, cell culture, inflammatory response

## Abstract

Peptide‐based hydrogels have attracted significant interest in recent years as these soft, highly hydrated materials can be engineered to mimic the cell niche with significant potential applications in the biomedical field. Their potential use *in vivo* in particular is dependent on their biocompatibility, including their potential to cause an inflammatory response. In this work, we investigated *in vitro* the inflammatory potential of a *β*‐sheet forming peptide (FEFEFKFK; F: phenylalanine, E: glutamic acid; K: lysine) hydrogel by encapsulating murine monocytes within it (3D culture) and using the production of cytokines, IL‐*β*, IL‐6 and TNF*α*, as markers of inflammatory response. No statistically significant release of cytokines in our test sample (media + gel + cells) was observed after 48 or 72 h of culture showing that our hydrogels do not incite a pro‐inflammatory response *in vitro*. These results show the potential biocompatibility of these hydrogels and therefore their potential for *in vivo* use. The work also highlighted the difference in monocyte behaviour, proliferation and morphology changes when cultured in 2D *vs.* 3D. © 2016 The Authors Journal of Peptide Science published by European Peptide Society and John Wiley & Sons Ltd.

## Introduction

In the past two decades, significant efforts have been made to develop novel biomaterials for a variety of biomedical applications such as regenerative medicine and cell therapy. One such class of material, which has attracted significant interest, is hydrogel as this soft, highly hydrated material can be engineered to mimic the cell niche to promote *in vitro* and *in vivo* tissue regeneration 1, 2, 3, 4, 5 or can be used as drug and cell *in vivo* delivery platforms. 6, 7, 8, 9 A variety of approaches can be used to design hydrogels, one such approach is self‐assembly of small molecules (low molecular weight hydrogelators – LMWH). One such class of LMWH are short synthetic peptides, which are of significant interest as they can be synthesised using standard chemical routes and therefore be obtained with high definition and high purity. In addition, being built out of natural amino acids, they can be designed to be biocompatible and biodegradable and can be metabolised by the body.^10^, ^11^, ^12^

**Figure 1:**
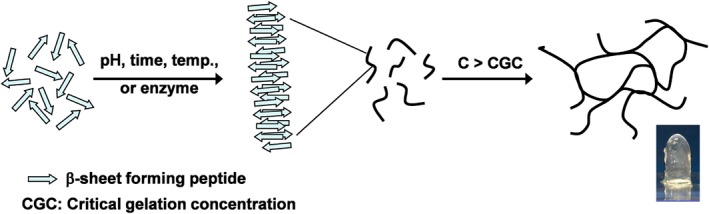
Schematic representation of the self‐assembly and gelation process of *β*‐sheet forming peptides.

A number of molecular designs have been developed for the synthesis of self‐assembling peptide LMWHs with the four main families being amphiphilic peptides, 13, 14, 15 short peptide derivatives, 16, 17, 18, 19, 20 *α*‐helix/coil‐coil peptides 21, 22 and *β*‐sheet peptides. 4, 23, 24, 25, 26, 27, 28, 29 *β*‐sheet peptides are of particular interest as they allow the fabrication of very stable hydrogels with properties that can be tailored through peptide design, media properties and processing. We have recently investigated the self‐assembly and gelation properties of a family of *β*‐sheet peptides 30, 31, 32, 33, 34 based on the design developed by Zhang and co‐workers. 23, 24, 25, 35 This design, which is based on the alternation of hydrophilic and hydrophobic residues, allows the synthesis of peptides that self‐assemble into anti‐parallel *β*‐sheet rich fibres. Above a critical gelation concentration, these fibres entangle and/or associate to form three‐dimensional networks that have the ability to trap water, i.e.: form hydrogels (Figure 1).

Biomaterial biocompatibility covers a variety of aspects including toxicity and biodegradability as well as inflammatory potential. In this work, we decided to focus on the investigation *in vitro* of the inflammatory properties of our hydrogel using monocytes. Monocytes are inflammatory cells that circulate within the bloodstream. In response to injury events, such as the implantation of a biomaterial, they migrate into the tissue/biomaterial and become macrophages. There are four subtypes of macrophages: M1, which have a pro‐inflammatory function; M2a, which have an anti‐inflammatory function; M2b, which are involved in immune‐regulation and M2c, which are responsible for tissue remodelling. 36 The presence of M1 promotes the necessary inflammatory response to protect the body initially after surgical implantation. 37, 38, 39 However, a prolonged M1 response ultimately leads to failure of the biomaterial to integrate. 40, 41 M1 macrophages respond to the pro‐inflammatory cytokine interferon‐γ and lipopolysaccharides (LPS), an integral part of gram‐negative bacterial membranes. In the presence of these signals, macrophages produce interlukin‐1*β* (IL‐1*β*), 6 (IL‐6), 12, 15, 18 and 23; tumour necrosis factor‐*α* (TNF*α*); chemokine ligand 15, 20 and *α*‐chemokine ligands 9, 10, 11 and 13. Monocytes can therefore be used as a marker of inflammatory response *in vitro*. Haines‐Butterick *et al.* tested the inflammatory properties of a 20 amino acid peptide hydrogel in 2D by seeding murine monocytes on the surface of the hydrogels and quantifying the production of TNF*α*. 42 They found minimal amounts of TNF*α* produced in response to their hydrogel scaffold. In our study, we decided to look at cytokine production after 3D encapsulation of monocytes in the hydrogel as this configuration is more representative of the *in vivo* situation, where the monocytes invade biomaterials. In our case, the production of three cytokines IL‐1*β*, IL‐6 and TNF*α* were quantified.

## Materials and Methods

### Monocyte Culture

Frozen murine monocytes (J774.2 Cell Line, Sigma–Aldrich, Dorset, UK) were cultured in DMEM (D6546, Sigma–Aldrich, Dorset, UK) containing 2 mM L‐glutamine (GE Healthcare Life Sciences, Buckinghamshire, UK) and 10% fetal bovine serum (FBS) (GE Healthcare Life Sciences, Buckinghamshire, UK). All cells were grown in an atmosphere of 5% CO_2_/95% air at 37 °C. Cells were dislodged into the media using a cell scraper prior to passaging. Cells were used at passage 5 in all experiments.

### Hydrogel Preparation

Hydrogels were prepared by dissolving ultraviolet sterilised FEFEFKFK peptide (Biomatik, Ontario, Canada; Purity 95% TFA salt) to a concentration of 20 mg ml^−1^ using a 30 : 70 mixture of sterile phosphate buffered saline solution (GE Healthcare Life Sciences, Buckinghamshire, UK) and sterile distilled water. After vortexing and overnight incubation at 80 °C, the peptide solution (pH 2–3) formed a hydrogel upon cooling at room temperature.

### Cell Encapsulation

To form the hydrogel within a ThinCert well, insert with 1.0‐µm pore size (Greiner Bio‐One Ltd, Gloucestershire, UK), 0.5 ml of hydrogel, prepared as described previously, was pipetted into the insert and placed within a 12‐well plate (Greiner Bio‐One Ltd, Gloucestershire, UK). The hydrogel pH was adjusted to 7 by adding 50 µl of a 0.5 M NaOH solution to the top of the hydrogel drop by drop and then gently mixing the NaOH in with the pipette tip (shear modulus of hydrogel after pH adjustment ~10 kPa.^2^). Cells were added to the top of the gel in 100 µl of cell suspension and mixed into the gel using the tip of the pipette to give a final encapsulated cell concentration of 2 × 10^5^ cells ml^−1^. Finally, 1 ml of complete media was added to the well plate around the insert containing the hydrogel before being placed in the incubator.

### Study Design

Table 1 describes the composition of the samples used in our study. The third negative control (NC3) was prepared as described previously, but media alone was added to the gel compared with media with cells in the test sample (TEST). For the positive controls, 100 µl of a 20 µg ml^−1^ LPS (Sigma–Aldrich) solution was mixed with the cells prior to plating the cells (PC1) or encapsulating the cells in the hydrogel (PC2). At 48 and 72 h samples' supernatant was removed and frozen at −80 °C for further analysis. The supernatant that was removed was replaced with an equivalent amount of complete media.

**Table 1:** Sample composition

Sample	Abbreviation	Cell number	Gel volume (ml)	Amount of LPS added (µg)	Volume of media added (ml)
Media only Negative control	NC1	No cells	No gel	0	1
Media + cell Negative control	NC2	2 × 10^5^ a	No gel	0	1
Media + gel Negative control	NC3	No cells	0.5	0	1
Media + cell + LPS Positive control	PC1	2 × 10^5^ a	No ge*l*	2	1
Media + gel + LPS Positive control	PC2	2 × 10^5^ b	0.5	2	1
Media + gel + cells Test sample	TEST	2 × 10^5^ b	0.5	0	1

a per 0.5 ml of hydrogel;

b added to well surface.

### Detection of IL‐1*β*, TNF*α* and IL6 by Enzyme‐linked Immunoabsorbent Assay

Samples were tested for the presence of pro‐inflammatory cytokines with enzyme‐linked immunoabsorbent assay (ELISA). All ELISA components were purchased from R&D systems (Abingdon, UK). Details are collated in Table 2. A seven‐point standard curve was generated for each cytokine tested using the supplied recombinant mouse cytokine in twofold serial dilutions and a standard blank of phosphate buffered saline.

**Table 2:** Specification of ELISA tests used

Cytokine	Capture antibody	Detection antibody	High standard (pg ml^−1^)
IL‐1*β*	Rat anti‐mouse IL‐1*β*	Biotinylated goat anti‐mouse IL‐1*β*	1 000
TNF*α*	Goat anti‐mouse TNF*α*	Biotinylated goat anti‐mouse TNF*α*	2 000
IL6	Rat anti‐mouse IL‐6	Biotinylated goat anti‐mouse IL‐6	1 000

### Statistical Analysis

A one‐way analysis of variance to determine statistical significance with a 95% (*p* < 0.05) and 99.5% (*p* < 0.005) confidence interval and post hoc Tukey tests were performed using ezANOVA software.

## Results and Discussion

**Figure 2:**
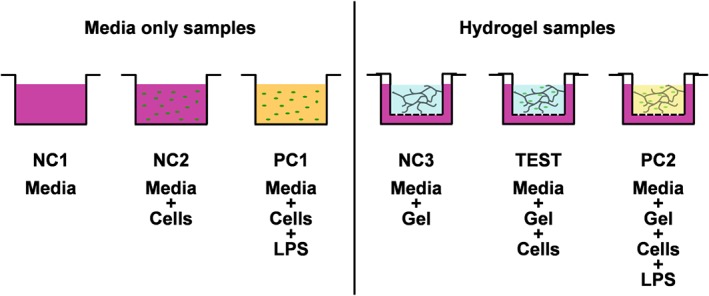
Experimental design. NC1: negative control 1 (media); NC2: negative control 2 (media + cells); PC1: positive control 1 (media + cells + LPS); NC3: negative control 3 (gel + media); TEST: test sample (gel + media + cells); PC2: positive control 2 (gel + media + cells + LPS).

In Figure 2, the experimental design used for this study is presented. In order to confirm the validity of the approach, a set of two negative (media and media + cells) and one positive (media + cells + LPS) controls with no hydrogels were prepared. These samples allowed us to confirm that the monocytes did respond to the presence of LPS. For the testing of the hydrogel in addition to the test sample (gel + media + cells), a negative (gel + media) control and a positive (gel + media + cells + LPS) control were prepared.

When dealing with hydrogels, ingress and egress via diffusion of large molecules such as LPS and proteins needs to be carefully considered. Diffusion of large molecules will depend on the hydrogel network properties (e.g. mesh size and charge) and the molecules' properties (e.g. aggregation propensity and hydrodynamic radius). In previous work, we have shown that the mesh size of our hydrogel is in the range of 20–30 nm.^31^ LPS are amphipathic molecules (~10–20 kDa), which in solution aggregate to form large structures (spherical and/or cylindrical micelles) with large hydrodynamic radii (~60–100 nm). 43, 44 On the other hand, interleukins and TNFα are found in solution in mono, di and/or trimeric states (~10–50 kDa) 45 and adopt compact conformations resulting in small hydrodynamic radii (~2–10 nm). We therefore expected LPS to be unable to diffuse into the hydrogels, while interleukins and TNFα were expected to be able to diffuse out. Preliminary tests indeed confirmed these assumptions. Neither interleukins nor TNFα were detected when LPS was simply added to the surrounding media of a hydrogel with monocytes encapsulated suggesting that the monocytes did not come into contact with LPS. On the other hand, when LPS was added to the cell solution prior to encapsulation into the hydrogels, interleukins and TNFα were detected in the surrounding media from 48 h onwards showing that the interleukins and TNFα were indeed able to diffuse out of the hydrogels. As a consequence of these findings, LPS was mixed into the hydrogels with the cells for the positive control sample, PC2. The production of interleukins and TNFα was measured by collecting the surrounding media and subsequently using ELISA as described in the Materials and Methods section. The detailed diffusive properties of these hydrogels have been the subject of a number of articles from our previous work^8^, ^34^ and work of other groups^6^, ^46^ and are beyond the scope of this article.

**Figure 3:**
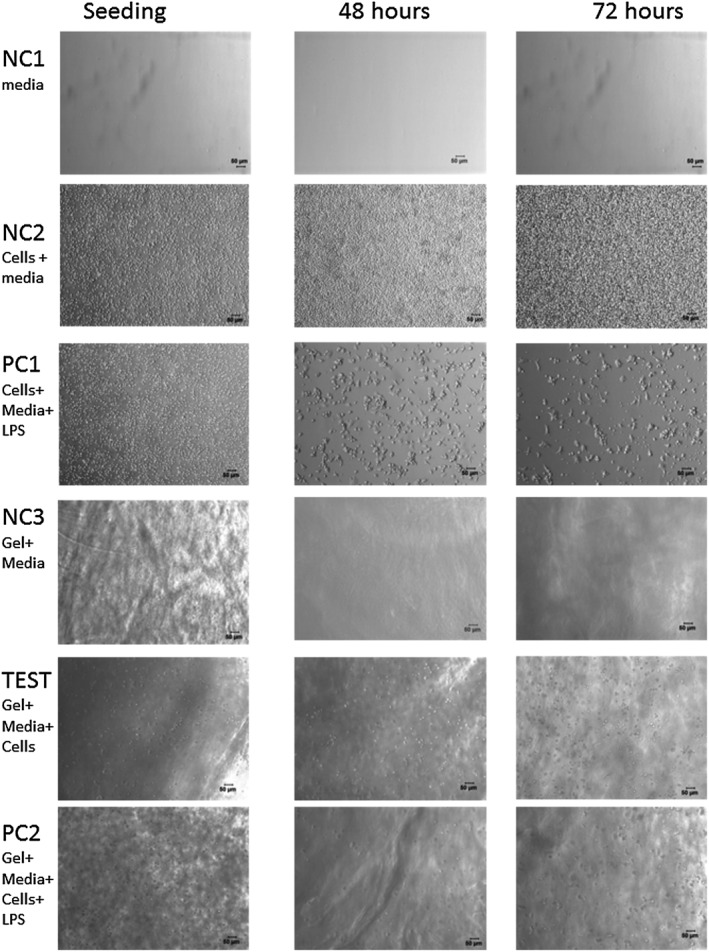
Light microscope images of samples at the time of seeding and after 48 and 72 h of culture. Scale bar represents 50 µm.

In Figure 3, light microscopy images obtained immediately after seeding the monocytes and after 48 and 72 h of cell culture are presented. As can be seen for the samples prepared without gel (2D culture), significant differences were observed between the negative control, NC2 (cell + media), and the positive control, PC1 (cell + media + LPS). For NC2, the monocytes were seen to proliferate rapidly, and after 48 h, they were already confluent, covering the full surface of the culture well. When LPS was added (PC1), the number of monocytes visibly decreased after 48 and 72 h of culture, and their morphology changed. Cells appeared larger and more extended suggesting that the monocytes responded to the LPS and differentiated into macrophages. When the monocytes were seeded in the hydrogel, the difference in morphology between the sample with LPS (PC2) and without LPS (TEST) was less obvious. Close inspection of the images seems to suggest a slight decrease in monocyte number, but no significant change in cell morphology was observed. As will be discussed next, for PC1 and PC2, a significant increase in cytokine level was detected suggesting that the monocytes were activated by the LPS in PC2 sample too. The difference observed in cell morphologies between 2D and 3D culture is not unexpected, indeed significant work in the literature has shown that cell behaviour and morphology can differ substantially when cultured in 2D or 3D. In our case, although the monocytes responded to the supplemented LPS even in the hydrogel, as will be shown next, there was no significant change in cell morphology probably because of the cells being encapsulated within a 3D matrix.

**Figure 4:**
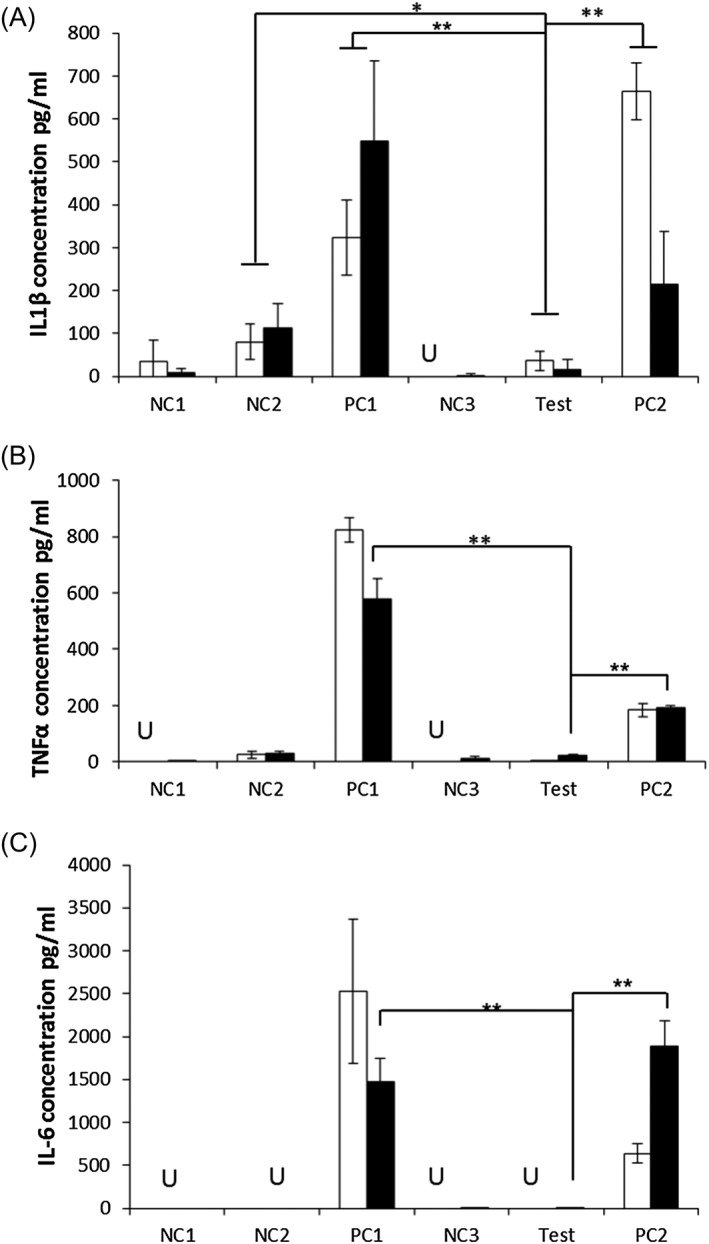
Amount of (A) IL‐1*β*, (B) TNF*α*, (C) IL‐6 detected in the different samples (see Figure 2 for details) in response to octapeptide hydrogel at 48 (white bars) and 72 h (black bars). Data points are mean of 6 replicates. Error bars represent standard deviation. U = undetectable; * *p* < 0.05; **p < 0.005.

In Figure 4, the amount of IL‐1*β*, TNF*α* and IL‐6 detected for each sample after 48 and 72 h of culture are shown. For IL‐6 and TNF*α*, significant and statistically meaningful levels of cytokines were detected only for the positive controls, PC1 and PC2. For IL‐1*β*, no statistical difference in interleukin produced between NC1 and NC3, and the TEST sample were observed. As NC1 and NC3 did not contain cells, this suggests that no statistically significant production of IL‐1*β* was detected in the TEST sample. On the other hand, a statistically significant amount of IL‐1*β* was detected in the two positive controls, PC1 and PC2. In this case though, statistically significant amounts of IL‐1*β* were detected in the negative control NC2, which contained cells. The increase of detectable IL‐1*β* in this sample can be explained by the rapid growth of these cells in a limited space, as shown by the light microscopy images, causing cell death and release of IL‐1*β*. This effect has been noted in another study where a lower seeding density was used. 42 Similar results to those seen for IL‐1*β* were observed for IL‐6 and TNF*α*. There was significantly more cytokine production in the two positive control samples, PC1 and PC2. There was no significant cytokine production detected in any of the negative controls; NC1, NC2 or NC3, or TEST samples. The lack of cytokines detected when the monocytes were encapsulated within our hydrogels suggests that they do not incite a pro‐inflammatory response *in vitro* making them a good candidate for use *in vivo*.

## Conclusions

We investigated *in vitro* the inflammatory potential of our peptide hydrogels by encapsulating murine monocytes within them (3D culture) and using the production of cytokines, IL‐*β*, IL‐6 and TNF*α*, as markers of inflammatory response. No statistically significant release of cytokines in our test sample was observed after 48 or 72 h of culture showing that our hydrogels do not incite a pro‐inflammatory response *in vitro*. These results show the potential biocompatibility of these hydrogels and therefore their potential for use *in vivo*. Our work also showed that 2D and 3D culture of monocytes in the presence or absence of LPS yielded very different cell behaviour. In the absence of LPS, the monocytes were found to proliferate rapidly in 2D while in 3D limited proliferation was observed. In both cases, the cell morphology remained unchanged. When LPS was added in 2D, limited cell proliferation and significant changes in cell morphology were observed, while in 3D, no significant changes in cell behaviour could be observed; although in both cases, an increase in cytokine production was observed confirming that in both cases, the monocytes were responding to the presence of LPS.
